# Sexual Relations as Causes of Uterine Disorders

**Published:** 1888-02

**Authors:** 


### *SEXUAL RELATIONS AS CAUSES OF UTERINE DISORDERS*

Is the heading of a timely and important article by Dr. William Goodell, of Philadelphia, in *Progress*, for October, 1887. The writer first speaks of the effects upon the female genital organs of the nervous erethism of long engagements. The love between man and woman is not merely an intellectual process, and the ante-nuptial relations sanctioned by custom in this country are the sources of diseases of which it is the duty of the physician to ascertain the cause and apply the remedy. The excesses of the honeymoon trip are to be deprecated. Counsel should be given regarding unmastered sexual excitement, while criminal abortion must be denounced. Scriptural and other authority are quoted to prove the sin of preventing conception, and a case is given, illustrating the harm resulting from intentional incomplete coitus. The various advantages of carrying out honorably, yet fully, the command to be "fruitful and multiply," and the serious mental and physical effects produced by any effort to baffle the laws of God, are pointed out in a manner that cannot fail to impress the reader.

